# Safety and efficacy of preoperative tranexamic acid in reducing intraoperative and postoperative blood loss in high-risk women undergoing cesarean delivery: a randomized controlled trial

**DOI:** 10.1186/s12884-022-04530-4

**Published:** 2022-03-14

**Authors:** Mohamed A. Shalaby, Ahmed M. Maged, Amira Al-Asmar, Mohamed El Mahy, Maged Al-Mohamady, Nancy Mohamed Ali Rund

**Affiliations:** 1 Department of Obstetrics and Gynecology, Kasr Al-Ainy Hospital, Cairo University, 366 Fardos gardens, 6 October, Giza, 12543 Egypt; 2 Department of obstetrics and gynecology, Ain Shams University, Cairo, Egypt

**Keywords:** Tranexamic acid, Elective CS, Intraoperative blood loss, Postpartum hemorrhage, High risk CS

## Abstract

**Background:**

Objective to assess the value of preoperative tranexamic acid (TXA) in reduction of intraoperative and postoperative blood loss in high-risk cesarean delivery (CD).

**Methods:**

A double blind randomized controlled trial included 160 high risk women who underwent elective lower segment CD. They were equally randomized to receive either 1 g of TXA or placebo 15 min before surgery. The primary outcome was Intraoperative blood loss.

**Results:**

The estimated blood loss was significantly higher in the placebo group when compared to TXA group (896.81 ± 519.6 vs. 583.23 ± 379.62 ml, *P* < 0.001).

Both postoperative hemoglobin and hematocrit were lower (9.2 ± 1.6 and 27.4 ± 4.1 vs. 10.1 ± 1.2 and 30.1 ± 3.4, *P* values < 0.001and 0.012 respectively) and their change percentages (15.41 vs. 7.11%, *P* < 0.001) were higher in the placebo group when compared to TXA one.

The need for further ecbolics was higher in placebo group when compared to TXA group (46.25 vs. 13.75%, *P* < 0.001).

**Conclusion:**

Preoperative TXA is safe and effective in reducing blood loss during and after high-risk CD.

**Trial registration:**

ClincalTrial.gov ID: NCT03820206.

## Synopsis

Preoperative administration of tranexamic acid is safe and effective in reducing intraoperative and postoperative blood loss during and after high risk CS.

## Introduction

Cesarean delivery (CD) is the most common major operation performed worldwide. The rates of CD increased from less than 10% before the 1980s to more than 30% in the last decade in many developed countries [1].

CD is associated with 2-fold increase in maternal morbidity compared with vaginal delivery [2].

Morbidities include infection, hemorrhage, thromboembolism, and anesthetic complications [3].

Obstetrical hemorrhage, hypertension and infection constitute the triad of maternal death causes [4].

Intraoperative and postoperative maternal hemorrhage are the main operative complications associated with high-risk CD. Anterior placenta previa, multiple pregnancies, and severe pre-eclampsia are all associated with a high risk of major PPH requiring immediate blood transfusion [1].

Many uterotonics as oxytocin, ergometrine and prostaglandins especially misoprostol were tested to minimize both intraoperative and postoperative. Bleed loss during and after CD [5].

Antifibrinolytic agents as tranexamic acid (TXA) were effective in prevention of bleeding complications with few side effects in various conditions [6]. WOMAN Trial Collaborators study proved that the use of TXA in women with postpartum hemorrhage had a large survival benefit [7]. TXA could decrease blood loss during surgery by almost 1/3 when compared to placebo [8].

TXA is a synthetic derivative of lysine with antifibrinolytic action. It blocks lysine binding sites on plasminogen molecules, preventing its interaction with formed plasmin and fibrin resulting in prevention of plasminogen activation with subsequent steadying of the preformed fibrin plug resulted from secondary hemostasis [9].

TXA was counted in WHO Model List of Essential Medicines [10] after confirmation of its ability to reduce mortality in trauma patients suffering from bleeding when administered early [11].

The use of TXA to reduce blood loss during and after surgery is routine nowadays in many procedures as coronary artery bypass, orthopedic and urological surgeries [12].

In obstetrics, TXA is used to treat pregnancy-related bleeding as threatened abortion, placenta previa and postpartum hemorrhage, [13].

Some studies proved the effectiveness of TXA in reducing blood loss during and after CD [14–16]. However, none of them targeted high risk CD.

The aim of our study is to investigate the safety and efficacy of preoperative TXA for the reduction of blood loss during and after elective lower-segment cesarean delivery to reduce intraoperative blood loss in high-risk lower segment cesarean sections.

## Materials and method

This study is a prospective, double-blinded, randomized placebo-controlled one that was conducted between January 31, 2019, and December 15, 2019 at Kasr alAiny maternity hospital, Cairo University. All participating women have signed an informed written consent after explaining the risks and benefits of the study. The consents were signed after approval of the kasr Alainy ethical committee. The trial was registered at clinical trial registry on January 29, 2019. NCT03820206.

All participants were scheduled for elective lower segment Cesarean section with their age ranged between 20 and 40 years old and gestational age between completed 37 and 41 weeks. All participants had one or more high risk for increased intraoperative blood loss. The risk factors included women with overdistended uterus (e.g. multiple gestation, macrosomic fetus > 4500 g or polyhydramnios with amniotic fluid index > 24), placenta previa, anemia and those who received intraoperative blood transfusion during prior CS.

Exclusion criteria included women with previous history of thromboembolic events, allergy to tranexamic acid and those with morbidly adherent placenta.

Women who were expected to encounter intraoperative complications as visceral injuries were also excluded from the study.

All participants were carefully evaluated through full history, general and abdominal examination to evaluate the risk factors properly and ensure adherence to our inclusion and exclusion criteria.

Obstetric ultrasound was done before surgery to assess the fetal age and maturity, placental location and amniotic fluid. Routine laboratory investigation were done including complete blood count and coagulation profile.

At the same surgical day, participants were equally randomized using computer-generated random numbers to one of the 2 groups. The anesthesiologist, obstetric surgeon, participants and outcome assessor were all blinded.

Fifteen minutes before surgery, women in the active group received 1 g (10 ml) of tranexamic acid (Kapron, Amoun, Egypt; diluted in 20 mL of glucose 5% while women in the control group received 30 mL of glucose 5%. Tranexamic acid ampoules were stored at 15–20 °C temperature in a dry contained. Both solutions were injected slowly over a period of 5 min [16].

All CS were done under regional anesthesia by obstetric surgeon with 5 or more years’ experience in obstetric management. The same technique was used in all women. Pfannenstiel incision, transverse lower segment uterine incision, Cord clamping immediately after fetal extraction, uterine exteriorization, two layers repair of the uterine incision and layered closure of the abdominal wall were done in all women. All women were followed up for 48 h.

After fetal extraction, all participants received a combination of intravenous 5 IU oxytocin (Syntocinon, Novartis, Basel, Switzerland) and intramuscular 0.2 mg ergometrine (Methergine, Novartis, Basel, Switzerland) followed by intravenous drip of 20 IU oxytocin diluted in 500 mL lactated Ringer’s solution with a rate of 125 mL/h).

All women have instructed to report any manifestations of thromboembolism. Reexamination for all participants was done after 1 and 4 weeks after discharge.

We calculated the intraoperative blood loss by taking the mean of the 2 famous methods of estimation. The first one was done through the formula Blood loss equals estimated blood volume (EBV) × preoperative hematocrit−postoperative hematocrit divided by preoperative hematocrit [17].

While the second one was through the weight difference of the dressings and towels before and after the operation added to the fluid volume inside the suction apparatus [5].

Intraoperative blood loss was the primary outcome parameter. Other outcomes included the need for further ecbolics, the need of intraoperative blood transfusion and occurrence of any side effects as thromboembolism.

Sample size was calculated using estimated intraoperative blood loss as the primary outcome. Elsedeek [18] reported the Mean ± SD postpartum blood loss as 324 ± 167 ml in the control group. Considering a 25% reduction in the blood postpartum loss, 80 women in the experimental group and 80 in the control were needed to reject the null hypothesis that the population means of the experimental and control groups are equal with probability (power) 0.9. The Type I error probability associated with this test of this null hypothesis is 0.05. Sample size calculation was done using G*Power software version 3.1.2.11.

Data were coded and entered using SPSS version 25 (IBM, Armonk, NY, USA). Data were described using mean ± SD, median, range for numerical data, and number and percentage for categorical data.

Kruskal-Wallis and Mann-Whitney tests were used to compare numerical variables and χ2 test was used to compare categorical data, *P* value less than 0.05 was considered statistically significant.

## Results

**Fig. 1:**
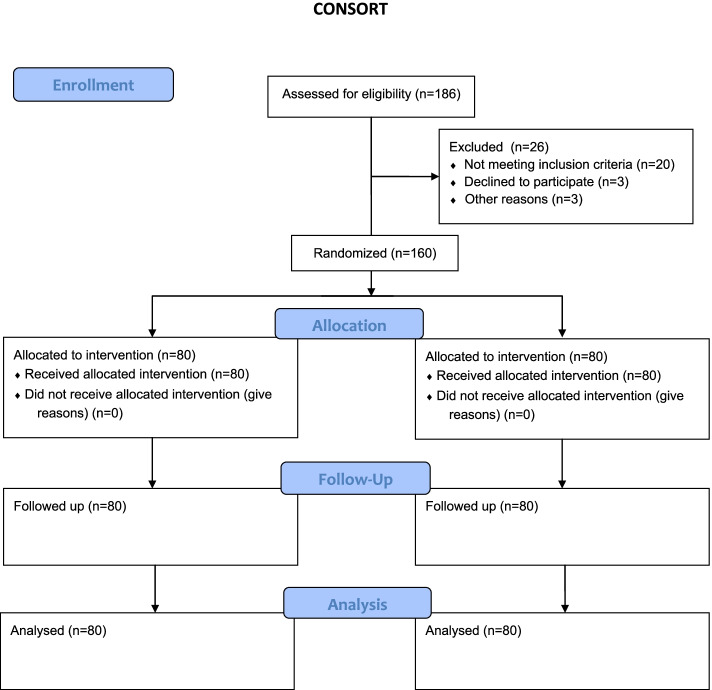
Consort flow chart

We assessed 186 women evaluated, 160 were randomized to one of the 2 groups (Fig. 1).

No statistical difference was found between women subjected to TXA and those subjected to placebo regarding maternal age, weight, gestational age or mode of previous delivery (Table 1).

**Table 1:** Demographic and clinical characteristics^a^

		Tranexamic acid group (*n*=80)	Placebo group (*n*=80)	*P* value
Age (years)		28.9 ± 4.46	28.5 ± 4.45	0.758
Weight (kilogram)		85.7 ± 7.8	90.1 ± 8.84	0.121
Gestational age (weeks)		38.1 ± 1.1	37.9± 1.1	0.729
Mode of previous deliveries	None	10 (12.5%)	12 (15%)	0.521
	VD	16 (20%)	19 (23.75%)	
	1 previous CS	34 (42.5%)	34 (42.5%)	
	2 previous CS	11 (13.75%)	10 (12.5%)	
	> 2 previous CS	9 (11.25%)	5 (6.25%)	
Risk factors	Anemia	11 (13.75%)	13 (16.25%)	0.623
	Polyhydramnios	8 (10%)	5 (6.25%)	
	Fetal macrosomia	15 (18.75%)	17 (21.25%)	
	Twin pregnancy	6 (7.5%)	9 (11.25%)	
	Placenta previa	31 (38.75%)	25 (31.25%)	
	Received blood transfusion during previous CS	9 (11.25%)	11 (13.75%)	
	Total	80	80	
Duration of operation (minutes)		49.9± 19.7	47.8± 19.1	0.341
Neonatal birth weight (grams)		3888.4 ±712.8	3912.1± 761.9	0.824
Neonatal outcome	Apgar 1 min	7.1 ± 0.9	7.0 ± 0.9	0.885
	Apgar 5 min	8.9 ± 1.1	8.9 ± 1.0	0.619
	NICU admission	8 (10%)	9 (11.25%)	0.662

^a^Values given as mean ± SD or number (percentage)

Placenta previa was the commonest risk factor in both groups followed by fetal macrosomia and anemia (Table 1).

The duration of the operation, neonatal birth weight, Parameters of neonatal outcomes named Apgar 1 min, Apgar 5 min and neonatal ICU admission were statistically not different between the 2 study groups (Table 1).

The estimated blood loss was significantly higher in the placebo group when compared to TXA group (*P* < 0.001) (Table 2).

**Table 2:** Estimated blood loss, hemoglobin, hematocrit, platelet count, and need for ecbolics^a^

	Tranexamic acid group (*n*=80)	Placebo group (*n*=80)	*P* value
Estimated blood loss (mL)^b^	583.23 ± 379.62	896.81 ± 519.6	<0.001
Hemoglobin concentration (g/dL)			
Preoperative	10.9 ± 1.1	11.0 ± 1.0	0.852
Postoperative	10.1 ± 1.2	9.2 ± 1.6	<0.001
Percentage change	7.34 (1.4–18.34)	16.36 (7.5–25.7)	<0.001
Hematocrit **%**			
Preoperative	32.8 ± 3.2	33.1 ± 3.0	0.662
Postoperative	30.1 ± 3.4	27.4 ± 4.1	0.012
Percentage change	7.11 (2.7–19.87)	15.41 (6.9–27.1)	<0.001
Need for further ecbolics	11 (13.75%)	37 (46.25%)	<0.001
Need for intraoperative blood transfusion	1 (1.25%)	5 (6.25%)	0.071

^a^Values are given as mean ± SD, median (range), or number (percentage).

^b^*EBL* Estimated blood volume (EBV)×preoperative hematocrit−postoperative hematocrit / preoperative hematocrit

Both postoperative hemoglobin and hematocrit were lower and their change percentages were higher in the placebo group when compared to TXA one (Table 2).

The need for further ecbolics was higher in placebo group when compared to TXA group (*P* < 0.001) (Table 2).

The need for intraoperative blood transfusion was more in placebo group compared to TXA one. However, the difference didn’t reach statistical significance (*P* 0.071) (Table 2).

## Discussion

The results of our study clearly demonstrated the ability of preoperative TXA to minimize intraoperative blood loss during high risk CD.

Placental separation during delivery is associated with powerful myometrial contractions, enhanced platelet activity, release of coagulation factors and increase of fibrinolytic activity (which continues for 6–10 h after delivery [19].

According to these facts, TXA can reduce blood loss after delivery regardless its mode through its fibrinolytic activity.

In our study, TXA significantly decreased intraoperative blood loss from 896.81 ± 519.6 in those who didn’t receive the drug to 583.23 ± 379.62 in women who received it.

Previous studies demonstrated the ability of TXA to decrease blood loss associated with CD.

Maged and colleagues in 2015 found in a RCT that 1 g of TXA could decrease blood loss in full term women who underwent elective lower segment CD from 700.3 ± 143.9 mL in control women to 459.4 ± 75.4 mL with only 6 participants had blood loss > 1000 mL. None of them was in TXA group. They didn’t report any side effects through 4 weeks follow up duration [16].

The reduction in blood loss was confirmed in another 2 studies [20, 21].

A large multicenter, double-blinded RCT, 4431 pregnant women who underwent CD were randomized to receive 1 g of TXA or placebo. Postpartum hemorrhage was reported in 26.7% (556/2086) and 31.6% (653/2067) in TXA and placebo group respectively adjusted risk ratio, 0.84; 95% confidence interval [CI], 0.75 to 0.94; *P* = 0.003). The rate of PPH was higher than the usual reported rates which may br related to the distribution of different risk factors. They concluded that TXA lowered the incidence of postpartum hemorrhage and the rate of red cell transfusion by day 2 but didn’t lower the hemorrhage related secondary outcomes named the use of additional uterotonic agents, and postpartum blood transfusion [22].

Both Sentürk and colleagues in 2013 [23] and Yehia and associates [24] confirmed the efficacy of TXA to reduce blood loss during CD without reporting any thromboembolic, gastrointestinal, or allergic complications. Similar findings were reported by Yang et al. [25] after vaginal delivery.

One meta-analysis included 104 studies confirmed the efficacy of TXA to decrease postoperative blood loss. However, that decrease was different according to the type of surgery and the time of TXA administration [8].

Another meta-analysis that included 34 articles only five of them were RCT and the rest was either observational (7) or case reports (22) also confirmed the efficacy of TXA to decrease blood loss. However, it reported pulmonary embolism in 2 cases without confirming the relation of these events to administration of TXA [26].

According to our findings, the percentage of change between preoperative and postoperative hemoglobin and hematocrit were significantly lower in women who received TXA when compared to control women (7.34 and 7.11 vs. 16.36 and 15.41 respectively).

In addition to the ability of TXA to decrease intraoperative blood loss, it markedly decreased the need for further ecbolics from 46.25 to 13.75%.

Similar decrease in the need for additional uterotonic drugs after administration of TXA was reported by Gungorduk and colleagues in 2011 [15].

We also demonstrated the ability of TXA to decrease the need of intraoperative blood transfusion from 6.25 to 1.25%. However, this difference didn’t reach a statistical significance. We believe that a larger sample size can detect a significant difference.

To the best of our knowledge, our study is the first RCT to evaluate the effect of TXA on blood loss during high-risk CD. It was double blind with properly calculated sample size. We believe that larger sample size could demonstrate additional benefits as need for transfusion. We used 2 methods to evaluate blood loss both the most common and most accurate methods. The main limitation of our study was the short follow up duration as the long term maternal and neonatal effects of the drug cannot be assessed.

The beneficial effects of TXA in reducing intraoperative and postoperative blood loss should be titrated against its side effects and risks. A future RCT with adequate sample size and long follow up is recommended to establish its value and whether it should be used routinely in high-risk women or in special population only.

## Data Availability

The datasets used and/or analysed during the current study available from the corresponding author on reasonable request.
