# Correction: Safety and efficacy of preoperative tranexamic acid in reducing intraoperative and postoperative blood loss in high-risk women undergoing cesarean delivery: a randomized controlled trial

**DOI:** 10.1186/s12884-022-05102-2

**Published:** 2022-11-07

**Authors:** Mohamed A. Shalaby, Ahmed M. Maged, Amira Al-Asmar, Mohamed El Mahy, Maged Al-Mohamady, Nancy Mohamed Ali Rund

**Affiliations:** 1 Department of Obstetrics and Gynecology, Kasr Al-Ainy Hospital, Cairo University, 366 Fardos gardens, 6 October, 12543 Giza, Egypt; 2 Department of obstetrics and gynecology, Ain Shams University, Cairo, Egypt

**Correction: BMC Pregnancy Childbirth 22, 201 (2022)**

10.1186/s12884-022-04530-4

Following publication of the original article [1], the sentence in the Methods beginning “Considering a 25% reduction …” should be replaced by the following:

Considering a 25% reduction in the postpartum blood loss, 100 women in the experimental group and 100 in the control were needed to reject the null hypothesis that the population means of the experimental and control groups are equal with probability (power) 0.95. Due to limited resources, recruitment was stopped after 160 participants had been included. Post-hoc sample size calculation using the same parameters showed that 80 participants in each group allowed for a power of 0.9.

The original article [1] has been corrected.
